# The Institutional Library

**Published:** 1918-01-05

**Authors:** 


### THE INSTITUTIONAL LIBRARY.

**The Principles of Rational Education.** By CHARLES A. MERCIER, M.D., F.R.C.P. (London: The Mental Culture Enterprise. 1917. Pp. 87+v. Price 2s. 9d. net.)

AMONG the *obiter dicta* which, as is his wont, Dr. Mercier lets fall in the discussion of his main theme, we read on page 7 of the present volume the announcement that "it is for the most part a sound rule of conduct to trust the expert." On the face of it this would seem to promise a free field for the professional teachers, as these surely may claim to be "experts" on education. But promptly comes the qualification. It is that when "the expert has failed conspicuously and egregiously in his own subject, we should receive his advice with hesitation and reserve." The schoolmasters and University pundits, according to Dr. Mercier, come under this condemnation, and hence he will have none of them, but will explore the subject for himself. His verdict is not an arbitrary one. The indictment is set out in plain terms, and the evidence duly displayed. Before us, standing in the dock, is a teaching profession which neither knows how to teach nor what subject should be taught, which cultivates a barren grind and a senseless routine, which is a slave to tradition and the dead hand, and which after years of opportunity sends its pupils out into the world deprived of originality and with heads "as empty as a drum." For the masters and mistresses of elementary schools this terrible judgment may in some measure be lightened, but for the Civil Service Commissioner, the University pundit, and the classical and clerical headmaster, there is reserved nothing less than the utmost terrors of the law. They must be broken on the wheel of public opinion, and after this discipline we may hope for new methods and new men. Parents and the public must provide the "moral dynamite" necessary to redeem the situation, and it is to these, therefore, and not to the scholastic experts, that Dr. Mercier addresses his pages. The experts must be treated as beyond the reach of repentance and as men wholly given over to idolatry. If all this, in substance, has been said before, we doubt if anywhere it has been said with such a force of argument or accompanied by such a volume of evidence. If the attack is a merciless one—as indeed it is—it is certainly not wanting either in courage or precision, and it is stated in the lucid phrases of which Dr. Mercier is an acknowledged master. He will not allow that any virtues which his writings may possess stand to the credit of his school or University training. We accept this disclaimer without reserve. His writings have many virtues—originality, sound reasoning, sincerity, good temper, a strict vocabulary, and a high standard of accuracy in expression. If the schoolmaster of his day gave him any training in any one of these, or even hinted to him that such achievements might be attained, then all we can say is that Dr. Mercier was much more fortunate than ourselves. His successes, we have not the slightest doubt, are due in part to the possession of what he himself would call a "faculty," and in part to the sustained and vigorous training of a voluntarily accepted self-discipline. We may all do something to improve our writings by imitating him in this latter respect, but the "faculty" is by no means a common possession. Here at all events men are not born equal, nor indeed can they by effort achieve equality. Some are, as it were, predestined to brilliant authorship, and others, let us say, to the writing of reviews. Thus for the literary landscape is secured the charm of variety.

From what has already been said it might be judged that Dr. Mercier's book is nothing but a wholesale and terrific onslaught on the traditional educational system and practice. This, however, is far from being the case. Indeed, the greater part of the book is devoted to an exposition of a method of training which is intended to replace the one now in vogue, supposing only that public and parental "dynamite" shall have secured free ground for the new instruction. Dr. Mercier's scheme must be followed in his pages. It is based on a study, first, of the material on which the educator has to work—that is, the child; second, of the result which the educator desires to produce; and third, of the means and method by which this result is to be brought about. The first of these sections includes a most engaging essay on the mental potentialities or "faculties" of the child, and upon this is founded a basis of differentiation in education when the stage of training of those "faculties" which are common to all children has once been passed. The truth of the rough proverb that "you cannot make a silk purse out of a sow's ear" has frankly to be faced, and one of Dr. Mercier's charges against the prevailing system is that this truth is largely ignored. In regard to the aim and purpose of education he puts "the imparting of knowledge" in but a subordinate place. The first aim is the development of character, the second a habit of clear thinking, and it is only in the third rank comes a "knowledge of facts." When these principles fall to be applied in actual practice it is at once evident that the method would involve a much more direct supervision of each individual child than the present system can even pretend to offer. And this would mean more expense, for it would imply that the number of scholars undertaken by each individual teacher must necessarily be strictly limited. Dr. Mercier recognises this, as he recognises also that if efficient teachers are to be secured the financial rewards of the profession must be substantially improved. We entirely agree. But what hope can be cultivated in this direction in an age which compels a daily expenditure of seven or eight million pounds on war and leaves hardly a living wage for those who are charged with the future hope and promise of the nation? Not yet have the ape and tiger been cast out or civilisation drawn itself clear from the barbaric past. A truly enlightened nation will demand the best minds for its schoolmasters, and especially, if we may say so, for its elementary schoolmasters. Here, of all directions, "faculty" is of supreme importance, and we could have wished that this supreme claim had been more strongly urged in Dr. Mercier's pages. Whether, when the new schoolmasters arrive and the "Promised Land" is gained, the system he advocates will be adopted, no one, of course, can pretend to say. But we feel quite sure that his book will always be recognised as a courageous attempt to improve our educational methods and thus to secure for the rising generation the opportunity to grow to the full stature of manhood. To read it is a truly enjoyable experience, and we should like to see it widely circulated among medical students. These have a valuable discipline within their grasp, but not all of them know how to use it. Nor do they always get much direction from their teachers, for in medical schools, even as in those of wider scope, the pundit and the pedagogue have a considerable representation.

### THE INSTITUTIONAL LIBRARY—(*continued*).

**Congenital Word-Blindness.** By James Hinshelwood, M.A., M.D. (London: H. K. Lewis and Co., Ltd. 1917. Pp. 112 + v. Price 4s. net.)

Dr. Hinshelwood has, by his original work on the subject of Word-Blindness, gained for himself such a position of authority that any contribution from his pen merits the highest consideration. It is some seventeen years since he published a work on the acquired form of word-blindness, and his arguments and conclusions have stood the test of time. He has continued his observations with fruitful results, as various papers in our current medical literature amply testify. The advance to the recognition of a congenital form of word-blindness is of great interest in itself, and it has associations with wide doctrines of cerebral function. We are convinced that Dr. Hinshelwood has done well in summarising his experience in the present volume and in restating the teaching which arises out of this experience. There are, apart from the question of convenient reference, two special reasons for this judgment. First, it is plain to those who follow our medical discussions on visual topics that not every speaker or writer has fully informed himself of Dr. Hinshelwood's views, and if he is to be quoted—as in such discussions he invariably is quoted—he naturally desires to be quoted correctly. A second reason for welcoming this book is that it relates to the training and education of a class of children—more numerous than is generally believed—who, while appearing stupid or obstinate under ordinary educational processes, are quite capable, by appropriate methods, of being fitted for the enjoyment and service of life. Congenital word-blindness, in short, is no high-and-dry topic remote from practical application. On the contrary, it demands recognition by all those charged with the education of children, and there is good ground for saying that the more widely such a possibility is known the larger the number of victims who will be identified. Dr. Hinshelwood has done excellent work in his studies of the subject, and we owe him additional thanks for the care and lucidity with which his teaching is expressed.

**Tropical Diseases.** A Manual of the Diseases of Warm Climates. By Sir Patrick Manson, G.C.M.G., M.D., LL.D., With twelve colour and four black-and-white plates and 254 figures in the text. Sixth Edition. Revised thoroughly throughout and enlarged. (London, New York, Toronto, and Melbourne: Cassell and Co., Ltd. 1917.)

Though the fifth edition of this manual was only published less than three and a-half years ago, the author has found it necessary to revise his text and to produce further illustrations. A few minor changes in arrangement have been made—viz., the chapter on pellagra is placed in the section of general diseases of undetermined nature, pace the vitamine school—and rat-bite disease in that of fevers. Colonel Alcock has revised the entomological part of the book and brought this up to date. The main text remains pretty much the same, and parts of this should really be re-written *in toto* if the book is to keep its place in the first rank of tropical works; if not, its popularity will wane, and eventually it will pass away in much the same way that the excellent works of Norman Chevers and others in India have done. It would benefit all tropical workers to have a good read of these Indian works on this important subject. For clinical details they will be found to be unrivalled, and the debt present writers owe to them will be readily appreciated after their perusal. The recent work on dysentery is not dealt with as fully as it should have been in Sir Patrick Manson's book—for example, the importance of the chronic carrier and the proper manner of treating such cases. Apart from these and other small points the book can still be recommended as a work worthy of careful study by the student and practitioner in the tropics.

**At the Serbian Front in Macedonia.** By E. P. Stebbing. (London: John Lane, The Bodley Head. Price 6s.)

The author of this somewhat diffuse and hastily written book served for some months as Transport Officer to a unit of the Scottish Women's Hospitals, leaving England in the early autumn of 1916. His experiences do not seem to have differed materially from those of many others whose fortune it has been during the last three years to visit the Balkan Peninsula on medical or military duty, and the chief value of the chronicle lies in the record of those little human touches which are apt to pass unnoticed in more pretentious volumes.

A hair-raising account is given of the adventures of the Ford ambulances (with girl chauffeurs!) upon the mountain tracks leading up to the great battlefield of Kajmaktcalan. "No Ford car brakes, which are necessarily light, would hold on these mountain tracks. The cars bumped down, now heeling over on one side, now on the other, as the wheels jolted over great masses of rock or boulders it was impossible to avoid, and on the steep slopes on many a journey the reverse was the only method of preventing the car taking charge when the brakes became functionless—and this with two badly wounded men on the stretchers behind." As most of the roads had no protection to save the unwary from falling headlong down a precipice, Macedonia will scarcely be a country to suffer from the iniquities of the road hog.

The book is illustrated by numerous photographs taken by the author, and its value is much enhanced by the addition of a map of the country and a consecutive account of the operations of the Allies from July to November 19, 1916, the date upon which Monastir fell.

**Glimpses of My Life in Aran.** Some Experiences of a District Nurse in these Remote Islands off the West Coast of Ireland. By B. N. Hedderman. Part I. Price 2s. 6d. net. (Bristol: John Wright and Sons.)

This uncommon little book is in some danger of being overlooked among more pretentious volumes. It has a real value, for it gives a picture, drawn with sincerity and good taste, of a part of the British Isles little known to any class of the public. Miss Hedderman undertook a task which called for absolute fearlessness and devotion to duty when she went to minister to the islands of Arran. The welcome they accorded her, though at first somewhat reluctant, is borne out in every line of her tale, but she has sternly set her face against gush, and the restraint of her narrative gives the reader confidence in her powers of observation and judgment. Her perils by sea are related as all in the day's routine, yet we think many a nurse would have been tempted to pose as a heroine after far less danger than she constantly encountered over an ordinary case. It would seem that her worst obstacles are not the winds and waves which torment her in her voyages by day and night from one island to another, but the deadly superstitions which still render the people an easy prey to disease and death. It is very pitiful to read of sick children neglected till they die because their parents are convinced they have been changed by the fairies.

